# Methodological Aspects of Obtaining and Characterizing Composites Based on Biogenic Diatomaceous Silica and Epoxy Resins

**DOI:** 10.3390/ma14164607

**Published:** 2021-08-17

**Authors:** Marta Dobrosielska, Renata Dobrucka, Dariusz Brząkalski, Michał Gloc, Janusz Rębiś, Julia Głowacka, Krzysztof J. Kurzydłowski, Robert E. Przekop

**Affiliations:** 1 Faculty of Materials Science and Engineering, Warsaw University of Technology, ul. Wołoska 141, 02-507 Warsaw, Poland; Marta.Dobrosielska@pw.edu.pl (M.D.); michal.gloc@pw.edu.pl (M.G.); janusz.rebis@pw.edu.pl (J.R.); krzysztof.kurzydlowski@pw.edu.pl (K.J.K.); 2 Department of Non-Food Products Quality and Packaging Development, Institute of Quality Science, Poznań University of Economics and Business, al. Niepodległości 10, 61-875 Poznań, Poland; 3 Faculty of Chemistry, Adam Mickiewicz University in Poznań, 8 Uniwersytetu Poznańskiego, 61−614 Poznań, Poland; d.brzakalski@gmail.com; 4 Centre for Advanced Technologies, Adam Mickiewicz University in Poznań, ul. Uniwersytetu Poznańskiego 10, 61-614 Poznan, Poland; julia.glowacka@amu.edu.pl; 5 Faculty of Mechanical Engineering, Bialystok University of Technology, Wiejska 45c, 15-351 Bialystok, Poland

**Keywords:** epoxy resin, diatomaceous earth, diatomite, silicone mould, glass mould, bio-composite, degassing

## Abstract

Diatomaceous earth are sediments of unicellular algal skeletons with a well-defined hierarchical structure. Despite many tests conducted on systems using diatomaceous earth and epoxy resins, we can find many differences in the methods of acquisition and characteristics of the composite, which may considerably affect the results. In our study, we have conducted tests to verify the impact of the method of obtaining samples and the degassing of the composite on its mechanical properties and standard deviation. The samples were cast in glass moulds and silicone moulds and then subjected to testing for their mechanical and functional properties, imaging with the use of an optical microscope and a scanning electron microscope. The tests have shown that, for samples cast in glass moulds, there is no heterogeneity within the area of the tested sample, as in the case of samples cast in silicone moulds. Silicone moulds allow for quite effective self-degassing of the resin due to the large area-to-mass ratio, and the small remaining air vesicles have a limited effect on the mechanical properties of the samples. The filler used also played a significant role. For systems containing base and rinsed diatomite, it is clear that the degassing of mixtures increases the tensile strength. For treated diatomite, the elongation at break grew along with increasing filler concentration, while for base diatomite, the improvement was observed for flexural strength and impact strength. A non-modified epoxy resin shows a tensile strength at 19.91 MPa (silicone mould cast). At the same time, the degassed, glass mould-cast systems containing 12% of base and rinsed diatoms showed a tensile strength of 27.4 MPa and 44.7 MPa, respectively. We have also observed that the higher the filler concentration, the higher were the tensile strength values, which for the rinsed diatoms reached over 55.1 MPa and for the base diatoms were maximum of 43.8 MPa. The tests, therefore, constitute a set of guidelines and recommendations for testing with the use of fillers showing an extended inner structure.

## 1. Introduction

Diatomaceous earth is an amorphous, naturally existing material with unique physical and chemical properties [1]. Moreover, it is a porous biological sedimentary rock consisting mostly of SiO_2_, with small quantities of Al, Fe, Ca, Na, Mg, and other elemental oxides as well as small organic impurities that fill its pores and are adsorbed on its surface [2]. Besides, raw diatomaceous earth contains non-defective structures, i.e., diatom frustules, as well as many structures that are defective, fractured, mechanically damaged and different in terms of external structural geometry, shape, and size. The literature describes methods of purifying diatomaceous earth.

From the practical point of view, deposits containing diatoms have the broadest application in composite materials. Despite a high spread of diatoms in the sea and freshwater, their acquisition directly from the environment is expensive and economically unreasonable. For this reason, the methods of extracting and purifying diatomaceous earth are an important direction in the use of that material to obtain composites. The most common method for large-scale production of diatomite is surface-mining of geologic formations containing fossilized diatomite layers [3]. Heavy equipment, including bulldozers and power shovels, is used. We know methods of purifying raw diatomaceous earth using thermochemical treatments with minimum calcination combined with acid treatment [4]. Based on these studies, it has been observed that a hot acid significantly affects the composition and structure of diatoms. Along with the increasing time and temperature, the natural diatomite was dissolving [5]. Raw diatomaceous earth was also subjected to chemical treatment with acids such as HF [6], HCl [7], or HNO_3_ [8]. The chemical treatment additionally allowed for removing contamination from diatomaceous earth, reducing the size of particles, and obtaining a material with a high SiO_2_ content. Yang et al. 2014 [9] used physical purification of raw diatomaceous earth with a laminar-flow centrifugal separator. Jung et al. [10] have developed a method to improve the porous structure of raw diatomaceous earth by using an electric field. The electric field allowed them to effectively remove contaminants from raw diatomaceous earth and to uncover any clogged and hidden small pores in the diatomite. Diatomaceous earth was also purified by rinsing with sodium hexametaphosphate (SHMP) used as a dispersant, in combination with centrifuging [11] or high-gradient magnetic separation (HGMS) to remove magnetic contaminants. In our previous tests [12], we used a method of fractioning and purifying raw diatomaceous earth by sedimentation, which allows for obtaining a fraction with a narrow distribution of diatom frustules. In our study, raw diatomite was mechanically mixed with water and fractionated with the use of sedimentation. This method, after modification involving the use of large-scale centrifuges, is economical on an industrial scale. The small, fractured frustule pieces were partly removed, along with bigger agglomerates/colonies, by suspending the diatomite in water and subjecting the suspension to sedimentation. Based on interacting gravitational forces, particles of different sizes were settling out at different ratios. The method we propose allows for producing a raw material with a more uniform design and morphology, which thus provides specific properties of the finished product. The proposed method makes it possible to refine diatomaceous earth and obtain economically added value, e.g., in the form of reduction of resistance during filtration or improvement of the properties of composite materials. The application of diatomite as a filler may not only reduce the price of epoxy composites and coatings but also improve their performance. Lee et al. reported epoxy resin composition containing titanium dioxide and diatomaceous earth to increase the hydrophobicity of the surface of coatings and provide them photocatalytic self-cleaning ability [13]. The application of mineral fillers such as diatomite is superior in terms of stability towards naturally occurring decay when compared to organic filler-based composites [14].

In this study, a mechanically fractionated raw diatomaceous earth was used as an epoxy resin filler. Among all polymers used, epoxy resins are not only widely used in adhesives, coatings, and the aerospace and electronics industries but also play an important role in marine engineering because of their easy processability, good mechanical and thermal properties, high chemical and corrosion resistance, good adhesion to various substrates, and low shrinkage on curing. However, the curing system has many shortcomings due to the high cross-linking density of the resin, such as inherent brittleness, poor toughness, and impact resistance. It is necessary to improve the above characteristics, especially the toughness, to apply the epoxy resin in the industry on a global scale for packaging, adhesives, paints, and coatings, [15,16,17,18]. In addition, despite the significant improvement of the already resins, composite materials based on the epoxy resin are still a subject of intensive research. Recent years have witnessed an increasing amount of research conducted to improve mechanical properties. However, the effect of different filler contents on the mechanical properties of epoxy resin polymers still requires further investigation, even though it is already known that the dispersion state, shape, and size of the filler could affect the inherent properties of epoxy resin [19].

Tests conducted on epoxy resin-based systems require the preparation of samples in the form of casts. Moulds made of silicone, steel, aluminium, or Teflon are widely used. Each of these materials has various advantages and disadvantages. Steel or Teflon moulds are more difficult to prepare and require more financial outlays, but they feature a lower wear and tear. Silicone moulds are made of relatively cheap materials, but they are less durable and repeatable. The type of mould used has the biggest impact on the dimensional compliance of the produced samples and the ease of casting and releasing the sample. In case of the limited possibility of proper resin degassing, the construction and orientation of the mould will affect the manner and location of the air bubbles forming in the sample. Moreover, the application of a silicone mould may result in the absorption of anti-adhesive (release) agents on the surface layer of the epoxy during casting, which affects its composition, surface appearance and properties, as well as other performance aspects of the final component [20]. From the point of view of designers and manufacturers of moulded components, the properties of the contaminated epoxy surface layer will be different to those of the bulk resin. In their work, Nogueira et al. prepared the tested epoxy resin-based systems with the use of silicone moulds [21]. They carried out mechanical tests that showed, among others, that the application of the said moulds causes standard deviations of tensile strength values ranging from 14.1% to 31.8%. Similarly, in a study [22] where silicone moulds were used to prepare the samples, the standard deviation was a maximum of 10.2%. In another study [23], composites were prepared with steel moulds. The authors have tested, for example, tensile strength, flexural strength, and flexural modulus. Standard deviations from the said values were approximately 6.3–24.6%, 5.1–10.1% and 4.75–9.7%, respectively. Similarly, in study [24], authors also used steel moulds, and the deviation from the flexural strength was around 7% and 0.75 GPa. In addition, study [25] covered the testing of mechanical properties of epoxy resin/ZrO_2_ composites, which were prepared using moulds made of aluminium. The deviation from the tensile strength was a maximum of 17.9%. In study [26], composites were prepared with moulds made of Teflon, and the deviation from the tensile strength was similar to that in other types discussed above (maximum of 14%). For this study, we have prepared resin/filler systems using conventional silicone moulds as well as glass moulds with CNC cutting of the samples.

## 2. Materials and Methods

### 2.1. Materials

For the tests, we used epoxy resins produced by Zakłady Chemiczne Organika Sarzyna: Epidian 601 with an epoxide equivalent of 0.50–0.55 mol/100 g and a viscosity of 700–1100 [mPa·s], cured with triethylenetetramine (curing agent Z-1). Diatomaceous earth (Perma-Guard, Bountiful, UT, USA) was derived from diatomite deposits.

### 2.2. Preparation of Samples

The tested composites of epoxy resin Epidian 601, cross-linked with amine-based curing agent Z-1, contained different quantities (0, 12, 24, 36, 48, 60, 70% vol.) of base and fractionated diatoms according to the methodology described in the previous study [12]. The tested composites of epoxy resin Epidian 601 (epoxy number [mol/100 g] 0.5–0.55, viscosity at 25 °C [mPa∙s] 800–1500), cross-linked with amine-based curing agent Z-1 (triethylenetetramine, viscosity at 25 °C [mPa∙s] 20–30; amine number [mg KOH/g] min. 1100), contained different quantities (0, 12, 24, 36, 48, 60, 70% vol., corresponding to 0, 2.6, 5.5, 8.0, 10.4, 12.7, and 14.5% by weight, accordingly) of base and fractionated diatoms with an average particle size distribution of approx. 10 µm. The choice of the resin/hardener system was dictated, on the one hand, by the necessity to obtain a low viscosity of the mixture, and on the other hand, by the optimal time of its final gelation. The epoxy resin with diatom frustules was mixed mechanically in a plastic vessel using a steel propeller (600 rpm, 10 min). The samples were cast in silicone and glass moulds. The glass moulds were used to obtain 4 mm thick panels which were then cut with a CNC milling cutter to produce profiles for testing. The test samples were prepared after three weeks of resin curing at ambient temperature. The test samples were prepared according to EN ISO 527-1 [27] and 178:2010 [28]. The tests were performed after three weeks of samples curing at 20 °C. Table 1 includes the sample designations used in the study.

### 2.3. Characterization Methods

For flexural and tensile strength tests, the materials obtained were printed into type 1B dumbbell specimens in accordance with EN ISO 527:2012 and EN ISO 178:2006. Tests of the specimens obtained were performed on a universal testing machine INSTRON 5969 with a maximum load force capacity of 50 kN. The traverse speed for tensile strength measurements was set at 2 mm/min, and for the flexural strength, it was also set at 2 mm/min. Charpy impact test (with no notch) was performed on an Instron Ceast 9050 impact machine(Instron, Norwood, MA, USA) according to ISO 179-1 [29]. The density of the composites obtained was determined with the hydrostatic method using a Sartorius YCP04MS analytical balance (Sartorius, Göttingen, Germany) at 20 °C with the use of distilled water. The morphology and microstructure of the prepared composites were observed by scanning electron microscopy. Samples in the form of powders and moulds were dusted with cupro-nickel using a GATAN duster. Observations were conducted with a Hitachi SU 8000 scanning microscope (Hitachi, Ltd., Chiyoda, Tokyo, Japan) with an accelerating voltage of 5 kV to image the preparations. The imaging was performed in three SE modes. Surface structure and breakthroughs were analysed under a Digital Light Microscope Keyence VHX 7000 with 100× to 1000× VH-Z100T lens (Osaka, Japan). All pictures were recorded with a VHX 7020 camera (Keyence International, Mechelen, Belgium). Differential scanning calorimetry was performed using a NETZSCH 204 F1 Phoenix calorimeter (Netzsch Group, Selb, Germany). Samples of 6 ± 0.2 mg were cut from each granulate and placed in an aluminium crucible with a punctured lid. The measurements were performed under nitrogen in the temperature range of 20–200 °C and at a 10 °C/min heating rate, and T_g_ was measured from the second heating cycle.

## 3. Results and Discussion

### 3.1. Influence of Gravity and Degassing

For the produced resin-based composite materials, filler sedimentation processes are important for particles exceeding 5 μm. Sedimentation is used to obtain quartz-epoxy conglomerates with a high filling rate. These effects were also observed for resin/diatomaceous earth systems. [12].

**Figure 1:**
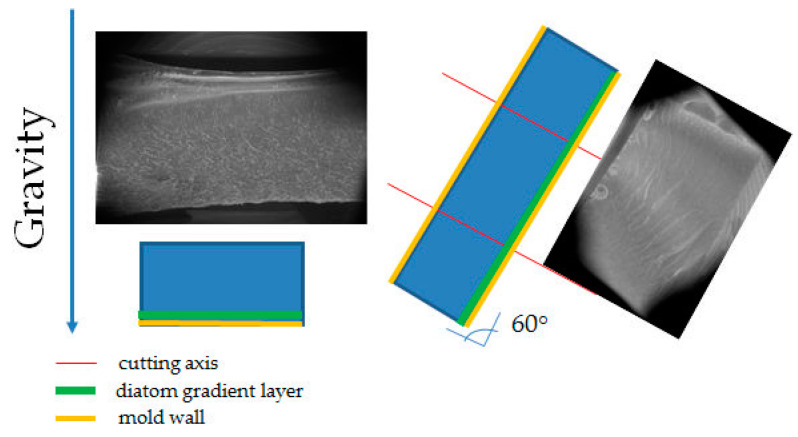
A glass mould, a silicone mould, inclination, and sedimentation direction of diatomaceous earth.

**Figure 2:**
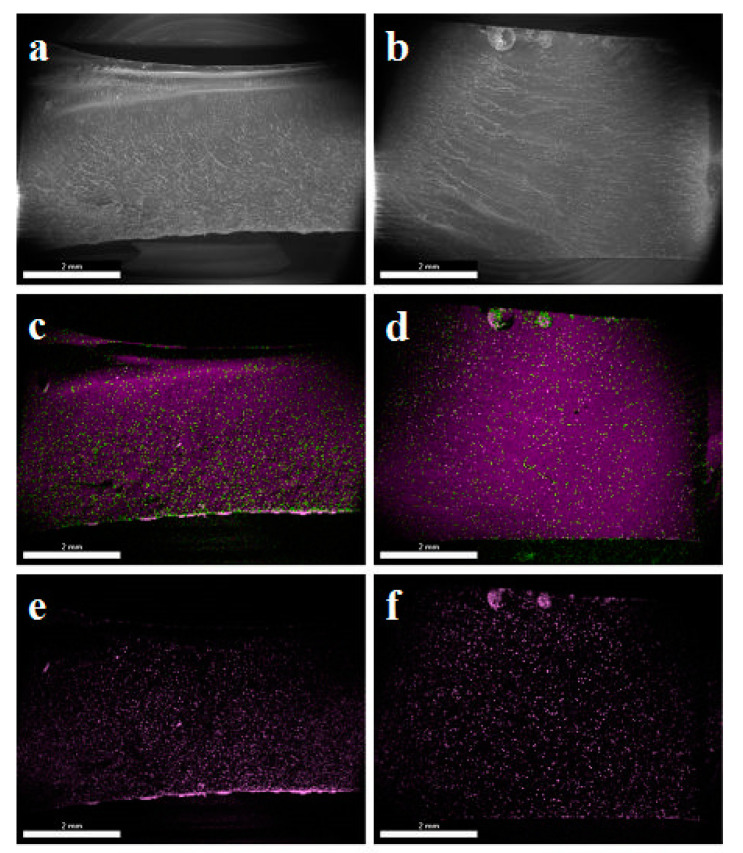
SEM/EDS images of diatoms/resin composites; (**a**,**c**,**e**)—image of a composite cast in a silicone mould. (**b**,**d**,**f**)—SEM image of a composite cast in a glass mould.

Figure 1 schematically shows sections of systems cast in silicone moulds and in glass moulds. The silicone mould during casting is positioned transversally to the gravitation direction (Z axis), so we can observe the concentration of mineral material in the lower part of the mould, with a proportional distribution towards the XY axes. For the glass mould, deviation from the XY plane was 60°. Such a position causes significant differences in filler sedimentation in the tested sample. If the glass mould was angled at 60°, this could cause a non-uniform concentration of the filler and the sedimentation being in line with the gravitation direction but not in line with the direction of CNC cutting. The tests have shown that, for samples cast in glass moulds, there is no heterogeneity within the area of the tested sample, unlike in the case of samples cast in silicone moulds. SEM/EDS images (Figure 2) show an even dispersion of diatomaceous earth in epoxy resin and the layout of filler particles being in line with the direction of CNC cutting. The systems cast in the silicone mould show an inclination to collect the filler closer to the bottom surface of the sample (Figure 2c), whereas the layout of particles in the samples cast in the glass shows that they are more homogeneous (Figure 2d). In addition, in non-degassed systems, we can observe floatation. Particles of diatomaceous earth are “entrained” along with air bubbles and gather by the top surface of the sample. (Figure 2d). This results in a slight accumulation of the filler in the upper layout of the composites.

**Figure 3:**
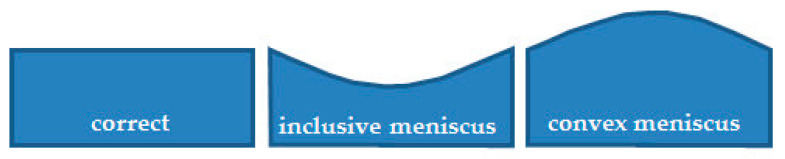
The issue of a convex and concave meniscus, casting in silicone.

**Figure 4:**
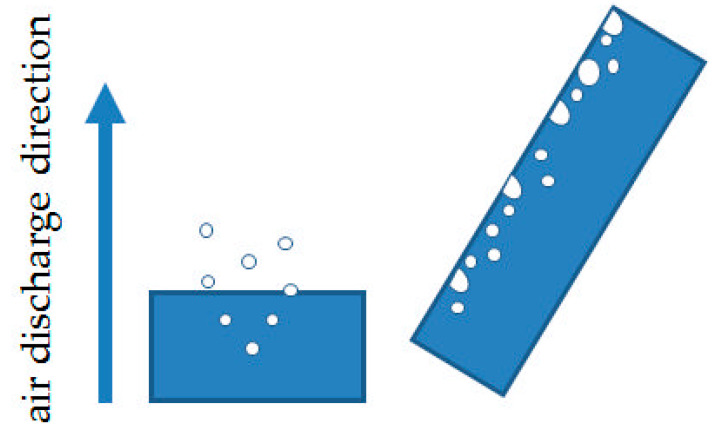
Degassing direction, a silicone mould on the left, and a glass mould on the right.

Another of the studied issues regarding the application of silicone moulds is the difference in the dimensions of test samples. Systems cast in silicone moulds are exposed to the meniscus effect (Figure 3), which does not occur in samples prepared in glass moulds. The meniscus is caused by misrun casting or, on the contrary, a slight casting-on of the top surface of the samples, which consequently leads to differences in the sample thickness being difficult to reduce and causes higher deviations in the values of the tested parameters. Samples cast in glass moulds feature constant dimensions, and their preparation by cutting out a measuring profile with CNC gives a dimension deviation lower than 0.03 mm. The SEM images show both a difference in composite thickness and the impact of the meniscus. Figure 2a clearly shows a concave meniscus produced by slight misrun casting of the sample, which causes non-heterogeneity of the samples in the series and a subsequent problem in testing mechanical properties, whereas the sample cast in a glass mould (Figure 2b) has its top surface flat, so the differences in dimensions between the samples in the series are minimal. The dispersion of diatomaceous earth in epoxy resin requires (intensive) mechanical mixing of constituents, which causes aeration of the system. Additionally, diatomaceous earth has internal voids of micrometric dimensions, filled with air. The choice of the mould type affects the degassing mechanism of the composite system. A silicone mould enables a free release of gas to the environment during gelation and cross-linking due to the relatively large sample-to-environment contact area (Figure 4). Any air bubbles that may remain in the samples are small and have a lower impact on mechanical properties. On the contrary, in the case of glass moulds, there are difficulties with air extraction. Gas bubbles accumulate on the mould’s top wall (according to the direction of buoyancy) and often merge into larger formations. An air bubble layer in a non-degassed sample might be 400 to even 900 µm thick depending on filler concentration.

A high content of air, which is difficult to remove given the structure of diatoms, causes the formation of untypical composites. Prior to cross-linking, a typical composite forms a three-phase system, i.e., it consists of a liquid phase (epoxy resin), solid-phase (powder filler), and gaseous phase (entrapped air), while after degassing and cross-linking it becomes a single-phase system that only consists of the solid phase. For composites with diatomaceous earth, we originally also deal with a three-phase system, but due to difficulties with extracting air, for example from the inside of diatom frustules, after cross-linking the system becomes two-phased: the solid phase is formed by a cross-linked, filled resin, and the gaseous phase, by air entrapped inside the composite, including in diatom frustules. A small amount of air is also present in pre-degassed systems. Such samples will show properties (for example, mechanical properties) different from those of single-phase composites.

**Figure 5:**
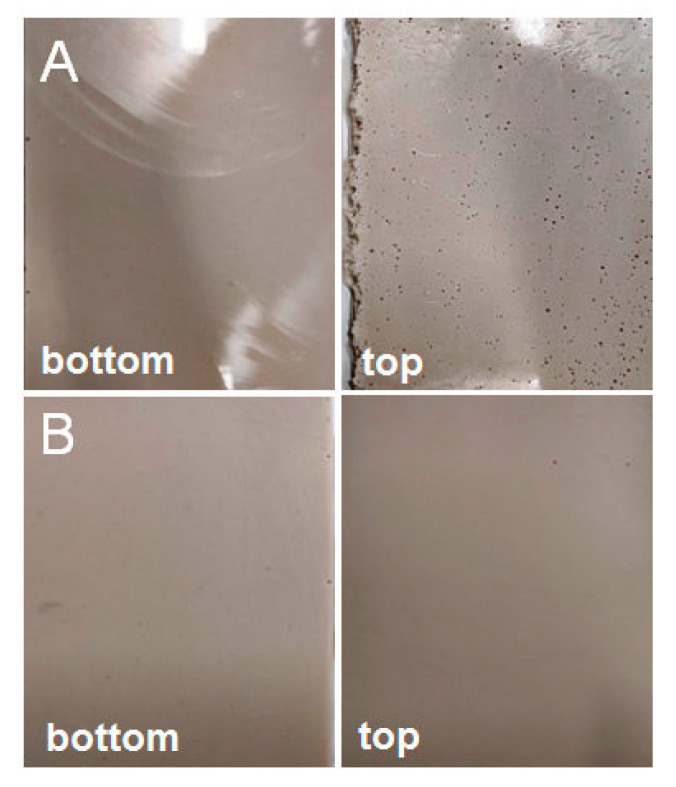
Panels cast in a glass mould, 70% of filling, (**A**)—without degassing, (**B**)—with degassing.

**Figure 6:**
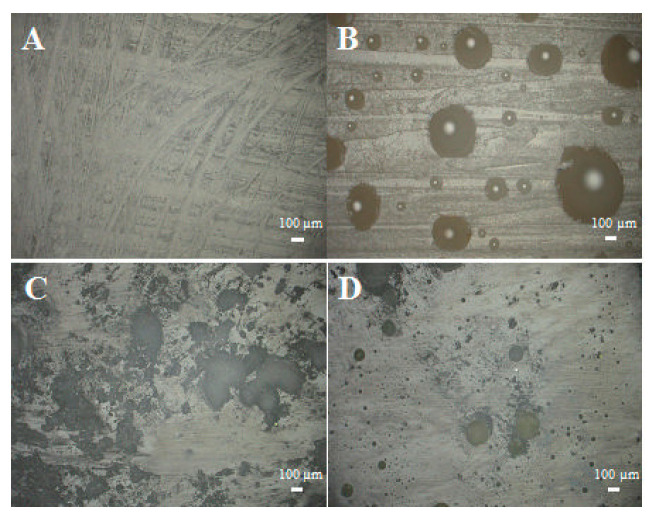
Photos of the samples’ surfaces, upper part, (**A**)—glass, degassed; (**B**)—glass, non-degassed; (**C**)—silicone, degassed; (**D**)—silicone, non-degassed.

**Figure 7:**
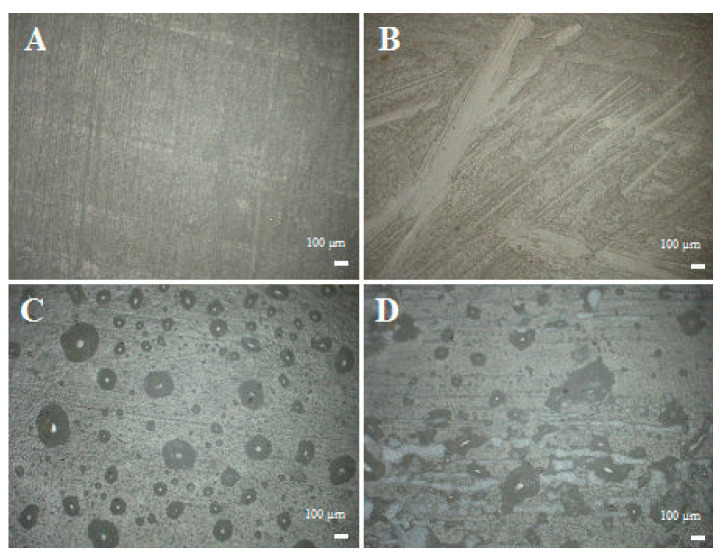
Surface of the samples: lower part. (**A**)—glass, degassed; (**B**)—glass, non-degassed; (**C**)—silicone, degassed; (**D**)—silicone, non-degassed.

**Figure 8:**
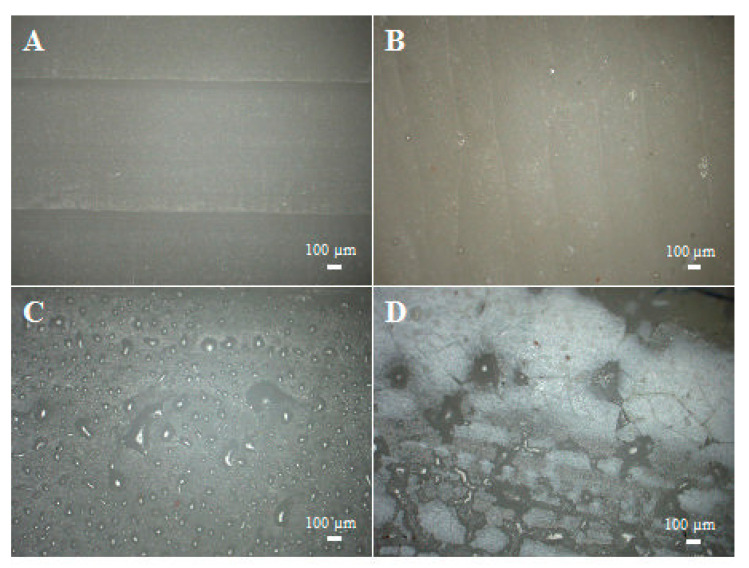
Photos of a side of the samples, (**A**)—glass, degassed; (**B**)—glass, non-degassed; (**C**)—silicone, non-degassed; (**D**)—silicone, degassed.

**Figure 9:**
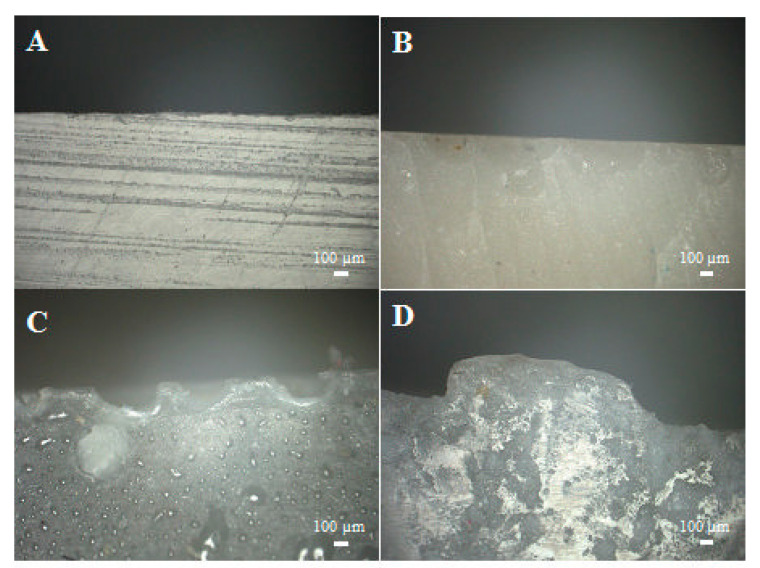
Photos of side edges of the samples, (**A**)—glass, degassed; (**B**)—glass, non-degassed; (**C**)—silicone, degassed; (**D**)—silicone, non-degassed.

As mentioned above, systems cast in glass moulds feature an untypical distribution of air bubbles. Figure 5a presents both the upper and the lower parts of an epoxy panel filled in 70% with diatomaceous earth without degassing. On the top surface, we can clearly see a layer of cavities caused by air bubbles, which do not exist on the bottom surface, where the panel is smooth. Figure 5b presents the system with the highest filling level, which was degassed before being cast into a mould. Both for the upper and the lower part of the panel, the cavities caused by air bubbles are virtually non-existent, which proves very good degassing and possibly better mechanical properties of the systems. The photos in Figure 6, Figure 7, Figure 8 and Figure 9 were taken to observe the surface structure and any defects in the composites. The photos in Figure 6 show the difference for the top surface of the samples cast in glass and in silicone, with and without degassing. The samples cast in glass and in silicone, which have been degassed, do not show any cavities caused by air bubbles, unlike the samples that have not been degassed. The large size of the cavities (Figure 6B) comes from the accumulation of bubbles by the surface.

Figure 7A–D present the bottom surface of the samples. We observed no difference between the samples cast in glass (Figure 7A,B); despite their different preparation (with or without degassing), their surfaces were almost identical. The air bubbles present in the non-degassed sample (Figure 7B) accumulated by the top surface, as mentioned above. The systems cast in silicone moulds (Figure 7C,D) also do not show any major differences in terms of the presence of air. Visible defects come from the reuse of the silicone moulds (missing fragments of the mould). The non-degassed sample (Figure 7D) contains partially embedded fragments of the silicone mould. The silicone mould, due to its short life and fast wear, adheres to the surface of composites, makes it difficult to remove the cross-linked sample, and, consequently, silicone fragments often remain on the bottom surface of the samples. This affects, for example, the hydrophobic and hydrophilic properties, as the removal of silicone before testing is hindered.

Figure 8A–D present the lateral section of the composites. The systems cast in glass must be cut with a CNC milling cutter prior to testing in order to obtain standardised samples. When a milling cutter moves across the sample, it leaves a characteristic mark after every plane of the milling path (Figure 8A). If the milled sample is not properly clamped during milling, and vibrations occur, the material gets chipped by the milling cutter (Figure 8B). The side wall of the samples cast in silicone includes cavities caused by mould defects (Figure 8C) but also many fragments being detached (Figure 8D). Both the degassed and non-degassed samples cast in glass moulds have a surface that is much more uniform than that of the samples cast in silicone moulds.

The photos of side edges (Figure 9A–D) of the samples allow us to obtain information on any defects that influence the systems’ properties. The degassed samples cast in glass have an almost completely even edge with no defects, whereas the non-degassed samples produced with the same method show small, shallow cavities by their edge, which are caused by the presence of air bubbles. The composites cast in silicone moulds also have more defects. These include deep cavities caused by air bubbles, silicone fragments (Figure 9C) and large projections (Figure 9D), which are caused by the wear of the silicone mould, so by cavities that form on it, into which the resin penetrates. The observed defects have a considerable influence on the mechanical properties of the tested samples, as described further in this paper.

### 3.2. Density

**Figure 10:**
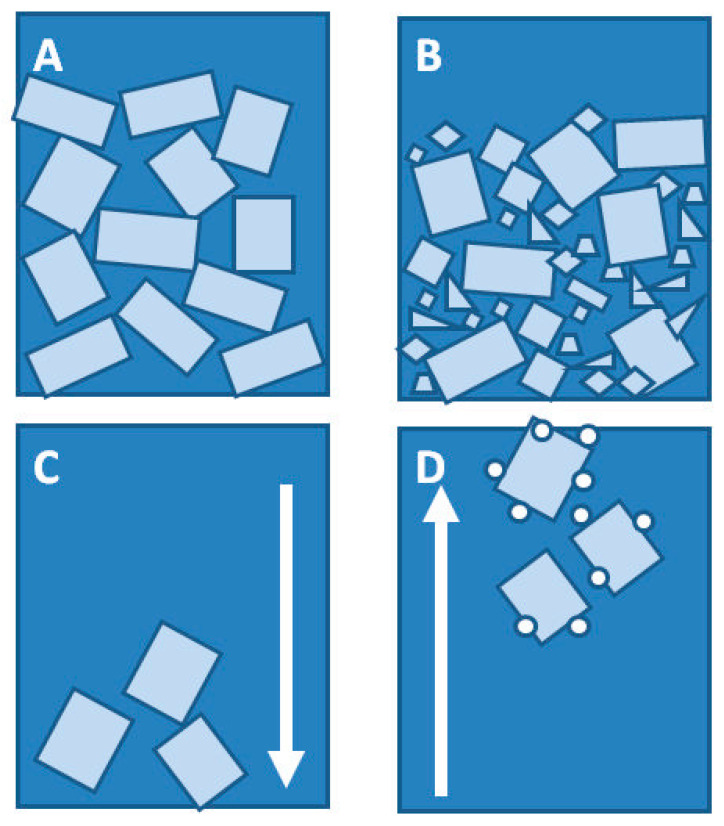
The mechanisms of packing and mobility of diatom frustules in an epoxy resin system. (**A**)—Manner of packing for a narrow particle size distribution, (**B**)—manner of packing for a wide particle size distribution (base diatoms), (**C**)—filler sedimentation in a resin, and (**D**)—filler floatation in a resin.

The fractioning of diatomaceous earth by sedimentation causes a change in particle size distribution in the system. Base diatomaceous earth contains particles ranging from 0.5 to 200 µm, which form fractured bubbles (<4 µm), non-fractured bubbles (4–50 µm) and agglomerates (50–200 µm) [12], whereas fractionated diatomaceous earth contains a small quantity of fractured particles and does not contain any particles bigger than 30 µm. For this reason, the packing of diatomite particles in epoxy resin is lower than in a fractionated system (due to the absence of fractions smaller than 5 µm). Any air remaining in the inner structure of a diatom is released by being replaced with resin, thus increasing the liquid phase aeration, which in turn hinders degassing in a vacuum chamber. In consequence, the higher the filler concentration and its packing in the resin, the higher the air content in the final product. Particles of diatomaceous earth may be subject to the sedimentation of epoxy resin and to floatation (Figure 10C,D).

**Figure 11:**
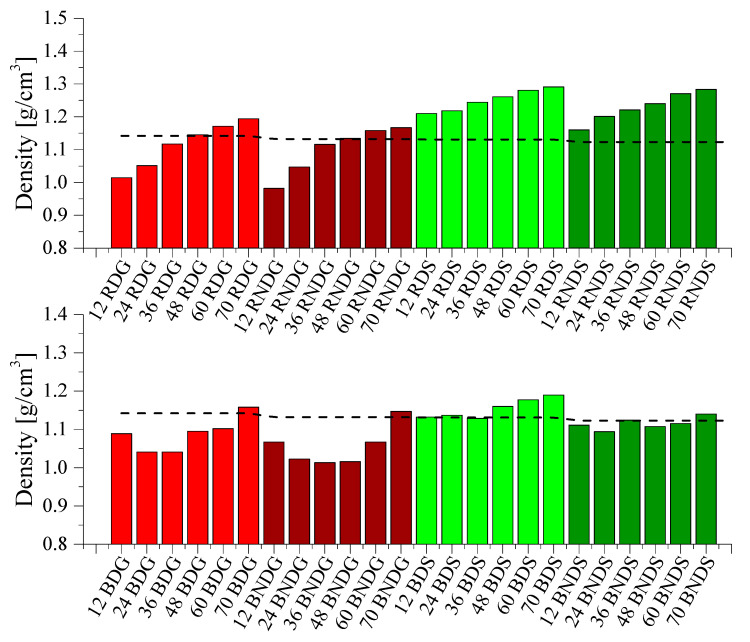
Density of degassed and non-degassed composites containing base diatoms and rinsed diatoms.

The bulk density of base diatoms is 0.271 g/cm^3^, and of rinsed diatoms is 0.248 g/cm^3^. A higher value for the base diatoms relates to the presence of particles of various sizes, which were removed from the second fraction during rinsing. Their presence increases the packing of particles and, consequently, reduces the air content between diatom frustules. The rinsed fraction consists of diatoms with more uniform sizes, between which more air is accumulated (Figure 11). Depending on their size, diatoms force a high quantity of air into the epoxy resin. The base fraction, due to its higher packing and the presence of smaller particles as well, causes lower aeration of the system, whereas the rinsed fraction contains larger spaces between bubbles and more uniform frustules, which causes higher aeration. Gas extraction from systems modified with base diatoms is more difficult given the formation of agglomerates, while rinsed diatoms after the same degassing time contain less gas. A higher quantity of air remaining in non-fractionated systems compared to fractionated systems causes a dilution of the effects related to filler content; the trend becomes flattened. At high filling levels, density is compensated for by the quantity of air entrapped. For silicone moulds, we can clearly see the secondary degassing effect.

### 3.3. Mechanical Properties

#### 3.3.1. Tensile Strength

**Figure 12:**
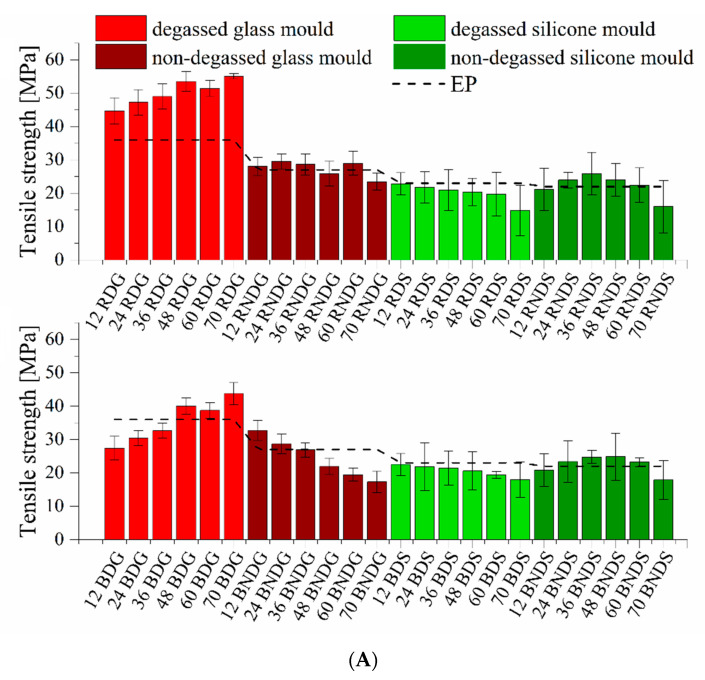

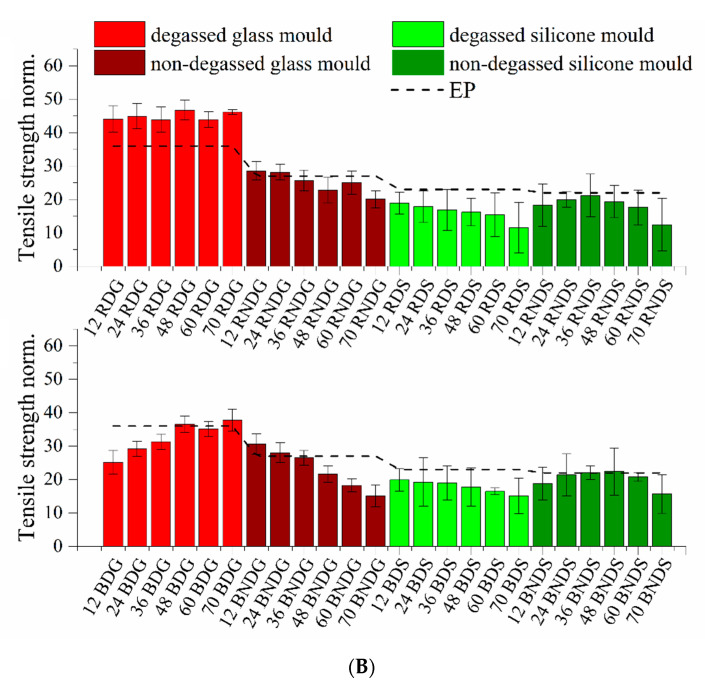
(**A**) Tensile strength for degassed and non-degassed composites containing base diatoms and rinsed diatoms. (**B**) Tensile strength for degassed and non-degassed composites containing base diatoms and rinsed diatoms, density normalized.

Both for systems containing base diatoms and rinsed diatoms, it is clear that the degassing of mixtures before casting into moulds increases the tensile strength (Figure 12A,B). That increase is clearer for base diatoms, even at lower concentrations. A non-modified epoxy resin shows a tensile strength at 19.91 MPa, whereas in almost any system containing diatomaceous earth that value is higher, sometimes over two-fold. In addition, the rinsing process also contributes to the growth of strength parameters. Degassed systems containing 12% of base diatoms and those after rinsing show a tensile strength of 27.4 MPa and 44.7 MPa. The higher is the filler concentration, the higher are the values, which for the rinsed diatoms reach over 55.1 MPa and for the base diatoms are maximum of 43.8 MPa. Composites cast in silicone moulds, due to their structural defects, feature lower strength characteristics. The changing trend as a function of filler content is generally linear. This indicates that defects in the sample prevail over the behaviour of the composite. The use of glass moulds allows for observing real dependencies for the characterised material. Difficulties with removing air from the composites also cause differences in the samples’ mechanical strength. Systems with base diatoms show a lower tensile strength than systems with rinsed diatoms, which is caused by the presence of a higher quantity of air in the base diatoms after degassing. Both the breaking strength and the elongation at break (see Figure S1) grow along with increasing filler concentration. A higher quantity of diatomaceous earth facilitates degassing, which reduces the brittleness of materials. If composites are not degassed before being cast into moulds, their mechanical properties are reduced, whether for base or rinsed diatoms. The reduction is proportional to the content of filler, which is caused by the growing number of air bubbles pressed into the systems by the diatoms. Gültürk, Taşdemirci et al. studied calcined and natural frustules filled epoxy matrices. The authors observed that debonding and pull-out may be the reason for the difference in mechanical properties when comparing neat epoxy resin and composites (the limited wetting of the filler surface by the matrix epoxy) [30,31].

#### 3.3.2. Flexural Strength

Flexural testing is considered an appropriate measure of strength because it combines elements of compression, tension, and shear, which more closely mimics in vivo stresses than either compression or tension testing alone [32]. Also, the produced composites were subjected to flexural testing with a three-point loading.

**Figure 13:**
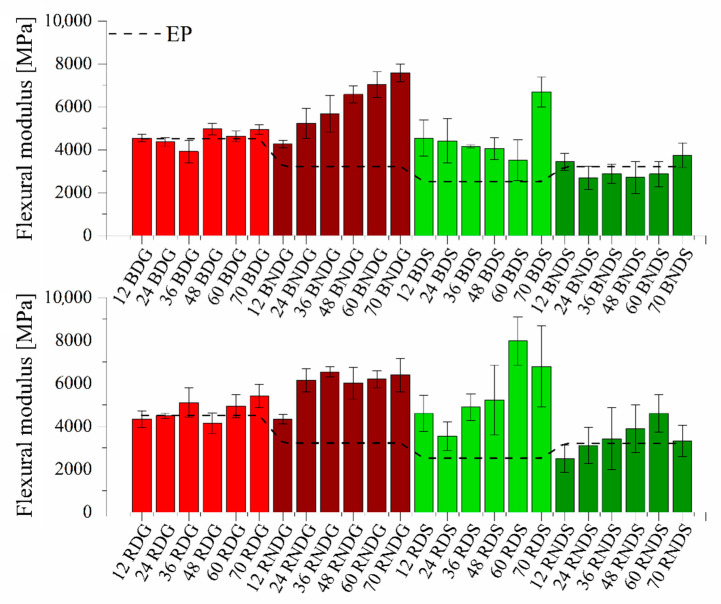
Flexural modulus for degassed and non-degassed composites containing base diatoms and rinsed diatoms.

Figure 13 present the flexural module and flexural stress of diatom/epoxy resin composites. Degassed systems cast in glass moulds, containing both base and fractionated diatoms, feature a flexural modulus ranging from 4 to 5 GPa. There is no impact of filler concentration on mechanical properties. Higher differences are observed in non-degassed systems cast in glass. Flexural modulus values grow in line with the growing filler content in the system, which applies in particular to the samples with base diatoms. For the highest concentration (70% vol.) of base diatoms, the flexural modulus is 7.5 GPa, whereas at the same concentration the value for fractionated diatoms is 1.2 GPa lower. Degassed systems show a lower air content, so their flexural modulus values increase. This is caused by a reduced brittleness of the samples. For systems cast in silicone moulds, this trend is reversed. Flexural modulus for the degassed samples increases along with the growing concentration of modifier, whereas for the non-degassed samples the values are similar in the entire series, regardless of the modifier used. An addition of diatomaceous earth, irrespective of its type, reduces flexural stress in each case (see Figure S2A,B) compared to a non-modified resin, which is caused by the presence of air bubbles in diatomaceous earth. Non-degassed systems show an elongation at break that drops in line with the growing modifier concentration, which relates to the fact that a higher quantity of the added filler contains more air entrapped in form of vesicles. Degassed systems show an opposite relation: flexural stress grows (up to 92.5 MPa) in line with the growing diatom concentration. As mentioned above, mixtures containing a higher quantity of modifier were easier to degas before cross-linking, thus the air content and, consequently, the brittleness of materials are lower. Systems cast in silicone moulds, whether degassed or non-degassed, have a similar flexural stress ranging from 27 to 61 MPa. We have neither observed any influence of filler type on flexural stress nor any presence of air in the systems.

#### 3.3.3. Impact Strength

**Figure 14:**
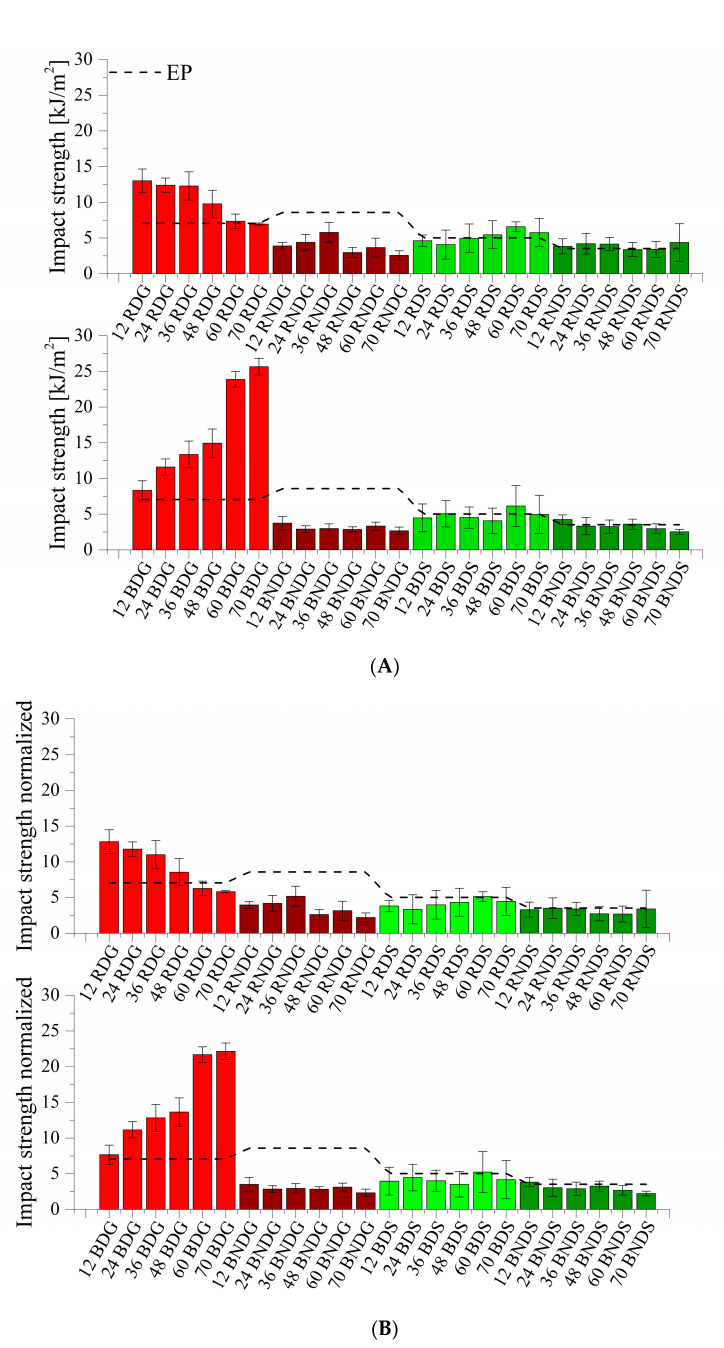
(**A**) Impact strength for systems containing base and fractionated diatoms. (**B**) Impact strength for systems containing base and fractionated diatoms, density normalized.

Figure 14A,B present the impact strength values. For most systems, it is similar and does not exceed 10 kJ/m^2^, whereas degassed systems cast in glass moulds feature higher values. Both the samples containing base diatoms and those containing fractionated diatoms show an impact strength of over 10 kJ/m^2^ up to nearly 26 kJ/m^2^. The addition of base diatoms caused a linear growth of the value along with the growing modifier concentration, whereas fractionated diatomaceous earth shows the highest impact strength for systems containing 12% vol. (13 kJ/m^2^); with a higher addition of modifier, the values linearly decrease. The differences in impact strength arise from the use of rinsing and from the degassing process. Systems containing air bubbles will have much lower impact strength values. The use of silicone moulds significantly contributes to the impairment of mechanical properties. Both degassed and non-degassed systems show, in most cases, impact strength values lower than that of a reference epoxy resin. It can be seen that, in this case, the deterioration of mechanical properties is not influenced by the filler type or the degassing of the systems prior to casting.

#### 3.3.4. Standard Deviations

**Figure 15:**
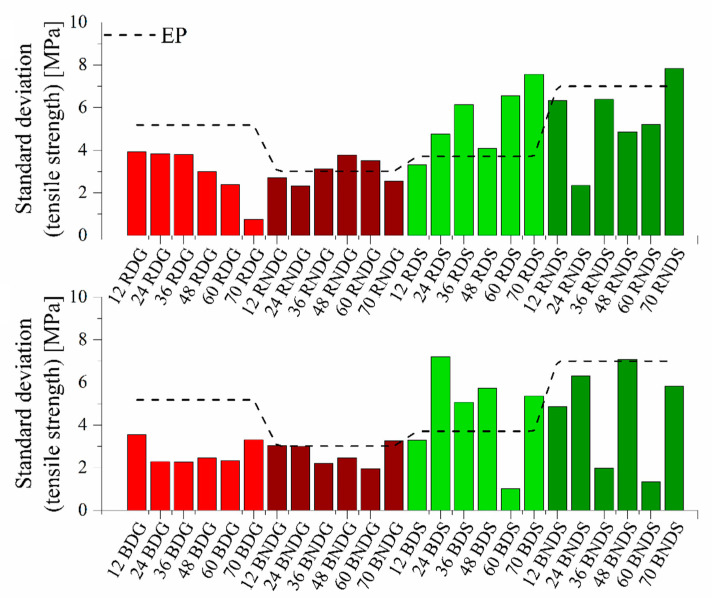
Standard deviations from the tensile strength value for systems containing base and fractionated diatoms.

As mentioned above, a number of factors have an influence on the uniformity of the results of mechanical property tests conducted on filled composites. Figure 15 presents the standard deviations from the tensile strength value.

Standard deviations for systems modified with base diatoms and rinsed diatoms fall within similar ranges. The maximum value of 4 MPa is reached by systems cast in glass moulds, while the value of 8 MPa is reached by a standard deviation for samples cast in silicone moulds. Rinsed systems cast in glass moulds, where degassing was used, indicate that the higher is the modifier content, the lower is the error bar, which is due to the higher homogeneity of material and the absence of defects caused by air bubbles. The values of standard deviation at the maximum elongation (see Figure S3) are much lower for systems containing base diatoms. This proves a better filler dispersion in the composite matrix. In addition, there are no significant differences between the deviation for samples cast in glass and in silicone moulds. Clearer differences exist in systems modified with rinsed diatoms. Higher standard deviation values exist for systems cast in silicone moulds.

**Figure 16:**
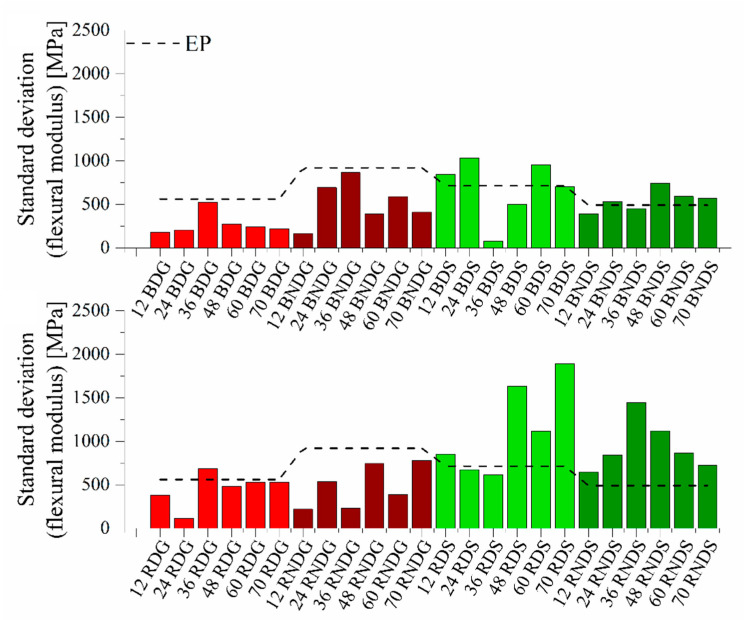
Standard deviations from the flexural modulus value for systems containing base and fractionated diatoms.

The standard deviations from the flexural modulus (Figure 16) for systems cast in glass moulds do not differ considerably between samples modified with rinsed diatoms and with base diatoms. In each case, the deviation is lower than that for a reference epoxy resin. Much higher deviation values exist for systems cast in silicone moulds (up to nearly 1900 MPa for 70% of rinsed diatoms), while an analogical sample cast in a glass mould shows a deviation of 530 MPa. This is similar to the results of the standard deviation in flexural stress (see Figure S4). Systems filled with base diatoms show lower deviations than those filled with rinsed diatoms. The standard deviation from the impact strength value (see Figure S5) is similar for systems modified with rinsed diatoms and with base diatoms. We can see a difference between the values for degassed and non-degassed samples. The values for non-degassed systems are lower, whether for glass or silicone moulds. Secondary agglomerates of the diatomite formed in the curing resin are a source of irregular mechanical defects (stress concentration centres or crack propagation sites), which result in unpredictable mechanical failure of the samples, which in turn causes an increased standard deviation. This secondary agglomeration effect is increased during degassing of the resin/filler compositions. This observation leads to a conclusion that diatomite should be initially properly surface-treated to improve the stabilization of its particles in epoxy compositions.

### 3.4. Thermal Analysis Results

**Figure 17:**
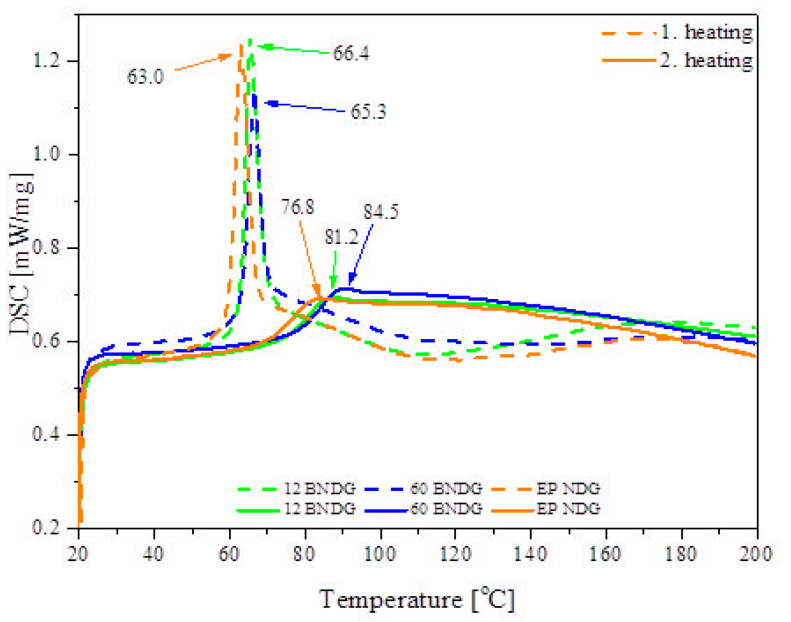
DSC curves recorded for samples of neat epoxy resin, and 12% and 60% BNDG.

DSC analysis was performed to study thermal behaviour of the composites (Figure 17). Two heating cycles were performed to register the most characteristic features of the thermal behaviour of the epoxy resin materials during their thermal treatment. In the first cycle, a sharp, low-temperature peak is observed at ~65 °C, which corresponds to the glass transition of the residually uncured resin (T_gu_). The residually uncured resin has much more polymer chain freedom due to lower cross-linking density when compared to fully cured resin, which results in a stronger endothermic effect. It can be seen that the diatomaceous earth/epoxy composites show slightly higher T_gu_ values, which can be explained by the fact that the filler acts as a heat insulator that delays the flow of heat into the samples. In the 80–160 °C range we can observe post-curing, as the remaining reactive epoxy and amino groups undergo a cross-linking reaction. During the second heating cycle, glass transition (T_g_) is observed at a higher temperature and with a significantly lower endothermic effect, which are both related to a reduced freedom of the cross-linked polymer chains. Again, a heat-insulating effect of the filler is observed, as the T_g_ of the diatomite composites is notably higher. This effect can be correlated with the filler loading, as the samples with a higher filling ratio showed higher temperatures.

## 4. Conclusions

The conducted tests were aimed to determine the impact of the manner of obtaining tests samples of epoxy/diatom frustule composites on the results of mechanical property measurements and on the existence of measuring errors. The tests have shown a significant impact of the applied method of casting test samples on a statistical dispersion of measuring results and on the mechanical properties obtained. The use of a silicone mould, given its relatively large area of contact with the environment, facilitates the degassing of any air that remains in the samples during cross-linking of the composite. At the same time, reusable silicone moulds cause defects on the test samples, which translates into repeating measurement errors. In the event of an improper degassing of the resin, the use of a glass mould angled at 60° causes sedimentation of air bubbles on the top surface of the composite and the formation of a porous layer that is 400 to 900 µm thick. Additionally, composites cast in a glass mould have a more homogeneous composition than those cast in silicone moulds, where the filler accumulates in the upper part of the sample. When using a specific filler such as diatom frustules (extended inner microstructure containing air), degassing issues grow along with the growing content of the filler in the resin. In such a case, complete removal of gas from the diatom frustule space for fast cross-linking resins is virtually impossible. The use of a silicone mould, unlike a glass mould, causes dimensional heterogeneity of the obtained composite samples, which also translates into measuring errors, and additionally, a (concave or convex) meniscus was observed, which had an impact on the low repeatability of dimensions of the samples and on measuring errors.

The systems produced in glass moulds showed even edges, constant dimensions and, consequently, a higher repeatability of results. The systems cast in glass moulds feature a higher tensile strength and elongation at break than the systems cast in silicone, which is probably due to (samples were non-coaxial or out of the plane of measurement) misalignment of the samples cast in silicone, which causes faster cracking (the impact of complex stresses on the sample). The tests constitute a set of guidelines and recommendations for testing with the use of fillers showing an extended inner structure.

## Figures and Tables

**Table 1:** Designations of the tested samples.

Nazwa Skrócona	Sample Name
EP	Reference
RDG	Rinsed with degassing glass mould
BDG	Base with degassing glass mould
RNDG	Rined without degassing glas mould
BNDG	Base without degassing glass mould
RDS	Rinsed with degassing silicon mould
BDS	Base with degassing silicon mould
RNDS	Rined without degassing silicon mould
BNDS	Base without degassing silicon mould

## Data Availability

Data available in a publicly accessible repository.

## Supplementary Materials

The following are available online at https://www.mdpi.com/article/10.3390/ma14164607/s1, Figure S1: Elongation at break for degassed and non-degassed composites containing base diatoms and rinsed diatoms; Figure S2: (**A**) Flexural stress for degassed and non-degassed composites containing base diatoms and rinsed diatoms; (**B**) Flexural stress for degassed and non-degassed composites containing base diatoms and rinsed diatoms, density normalized; Figure S3: Standard deviation at the maximum elongation for degassed and non-degassed composites containing base diatoms and rinsed diatoms; Figure S4: Standard deviation at the flexural stress for degassed and non-degassed composites containing base diatoms and rinsed diatoms; Figure S5: Standard deviation at the impact strength for degassed and non-degassed composites containing base diatoms and rinsed diatoms.
