# Supplementary material for: Methodological Aspects of Obtaining and Characterizing Composites Based on Biogenic Diatomaceous Silica and Epoxy Resins
Source: Materials (Basel). 2021 Aug 17;14(16):4607. doi: 10.3390/ma14164607 (PMC8400890; doi:10.3390/ma14164607)
Supplement: Supplementary file 1 [file materials-14-04607-s001.zip › materials-1293243 supplementary.pdf]

materials

MDPI

Article

Methodological Aspects of Obtaining and Characterizing Composites Based on Biogenic Diatomaceous silica and Epoxy Resins

Marta Dobrosielska [1], Renata Dobrucka [1,2,*], Dariusz Brząkalski [3], Michał Gloc [1], Janusz Rębiś [1], Julia Głowacka [4], Krzysztof J. Kurzydłowski [1,5] and Robert E. Przekop [4,*]

1. Faculty of Materials Science and Engineering, Warsaw University of Technology, ul. Wołoska 141, 02-507 Warsaw, Poland; Marta.Dobrosielska@pw.edu.pl (M.D.); michal.gloc@pw.edu.pl (M.G.); janusz.rebis@pw.edu.pl (J.R.); krzysztof.kurzydlowski@pw.edu.pl (K.J.K.)
2. Department of Non-Food Products Quality and Packaging Development, Institute of Quality Science, Poznań University of Economics and Business, al. Niepodległości 10, 61-875 Poznań, Poland
3. Faculty of Chemistry, Adam Mickiewicz University in Poznań, 8 Uniwersytetu Poznańskiego, 61−614 Poznań, Poland; d.brzakalski@gmail.com
4. Centre for Advanced Technologies, Adam Mickiewicz University in Poznań, ul. Uniwersytetu Poznańskiego 10, 61-614 Poznan, Poland; julia.glowacka@amu.edu.pl
5. Faculty of Mechanical Engineering, Bialystok University of Technology, Wiejska 45c, 15-351 Bialystok, Poland

* Correspondence: Renata.Dobrucka@pw.edu.pl (R.D.); r.przekop@gmail.com, rprzekop@amu.edu.pl (R.E.P.)

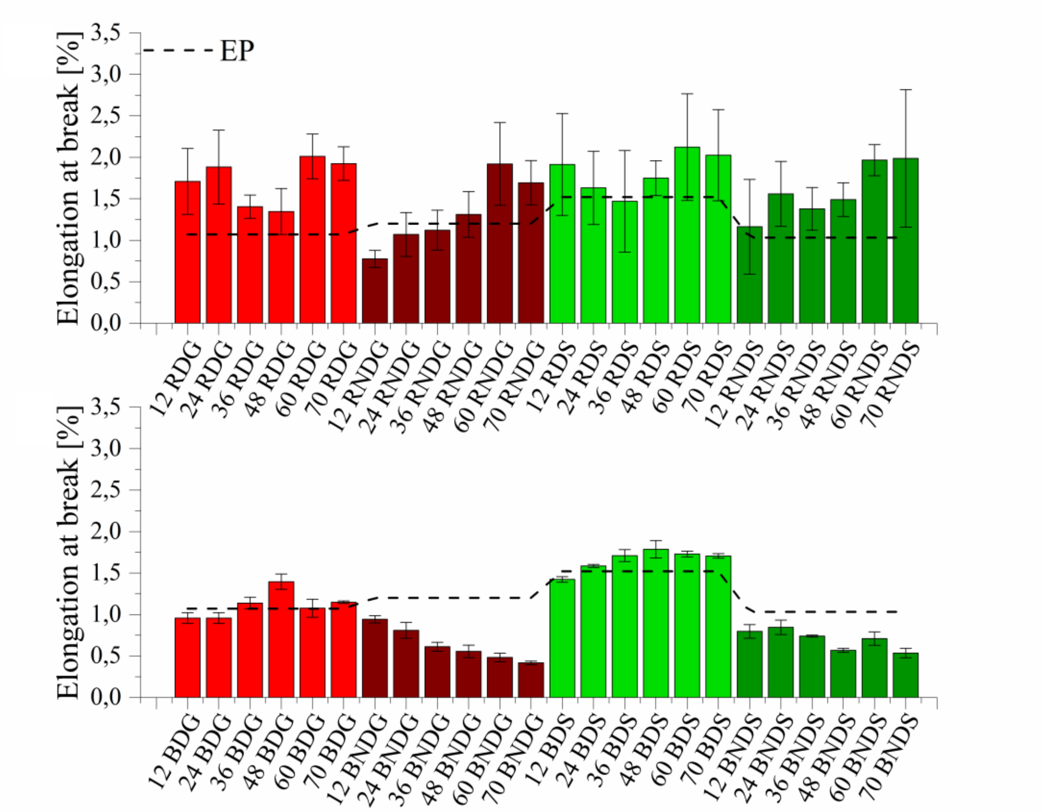

Figure S1. Elongation at break for degassed and non-degassed composites containing base diatoms and rinsed diatom.

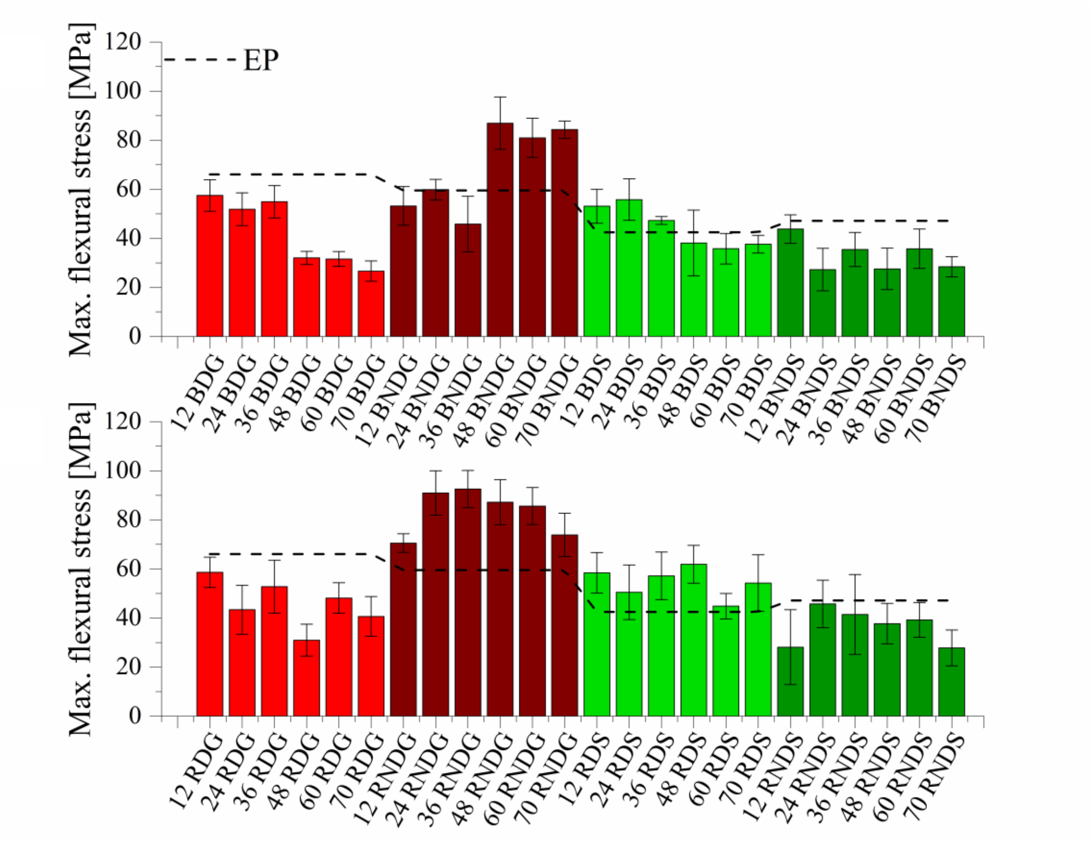

(**A**)

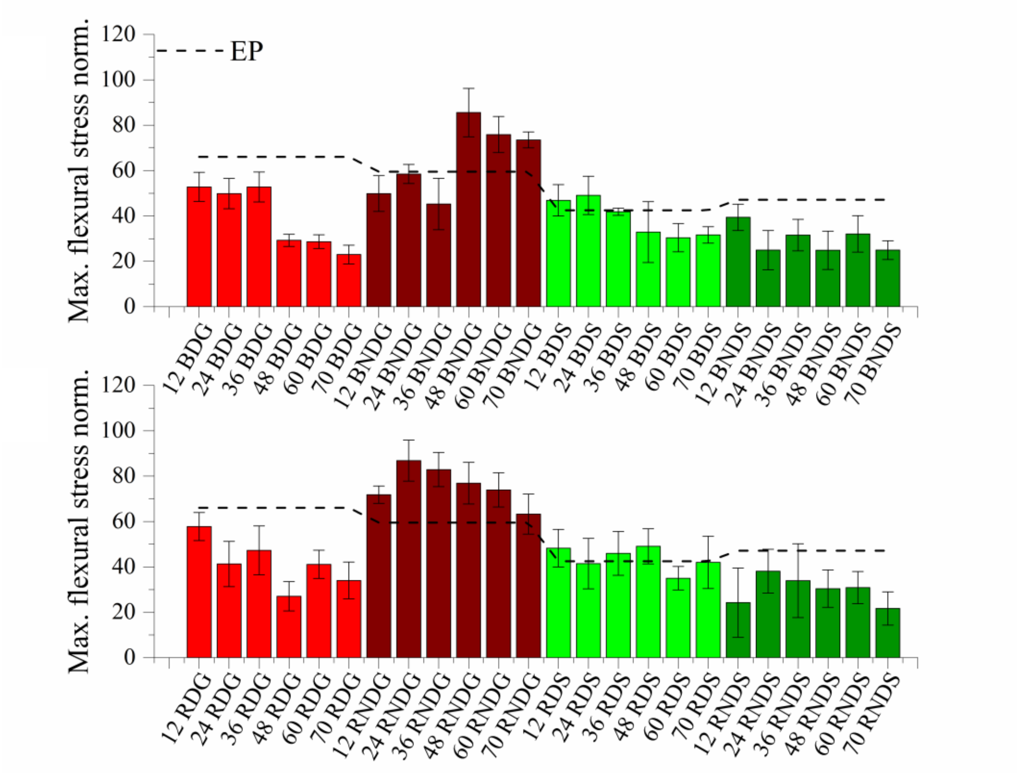

(**B**)

Figure S2. (**A**) Flexural stress for degassed and non-degassed composites containing base diatoms and rinsed diatoms; (**B**) Flexural stress for degassed and non-degassed composites containing base diatoms and rinsed diatoms, density normalized.

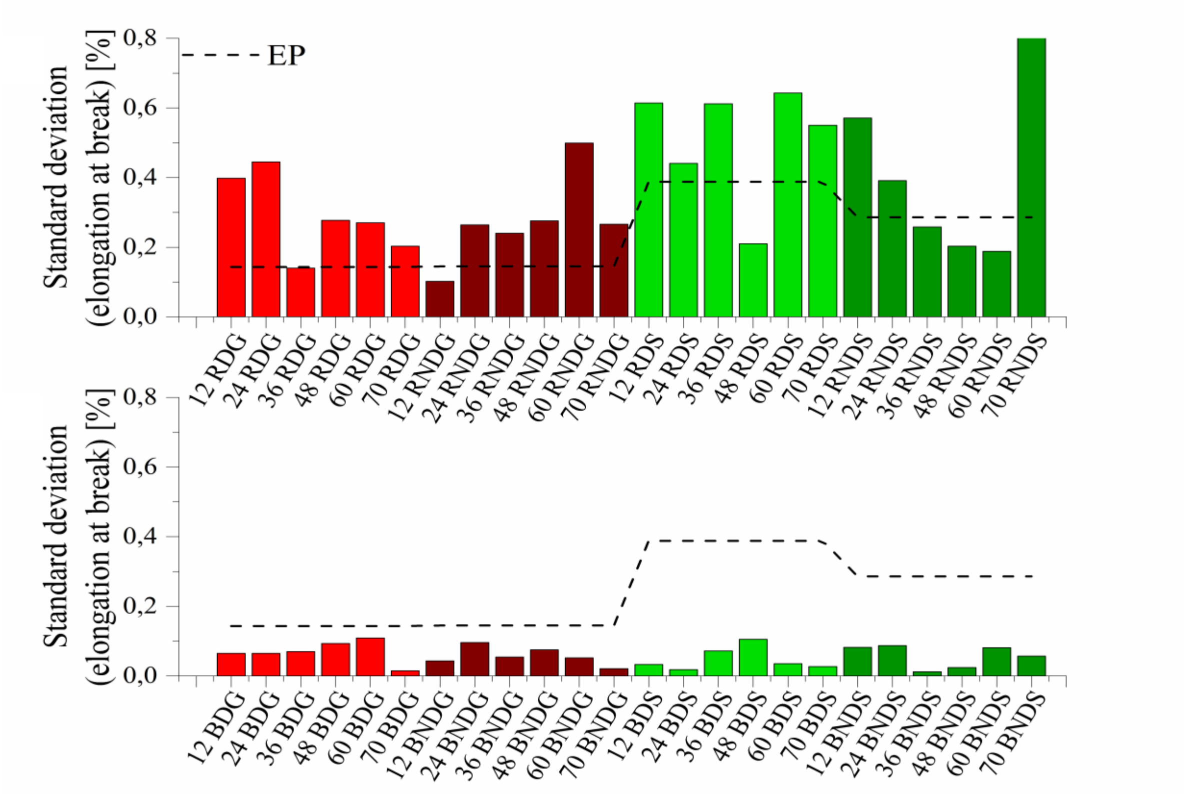

Figure S3. Standard deviation at the maximum elongation for degassed and non-degassed composites containing base diatoms and rinsed diatoms.

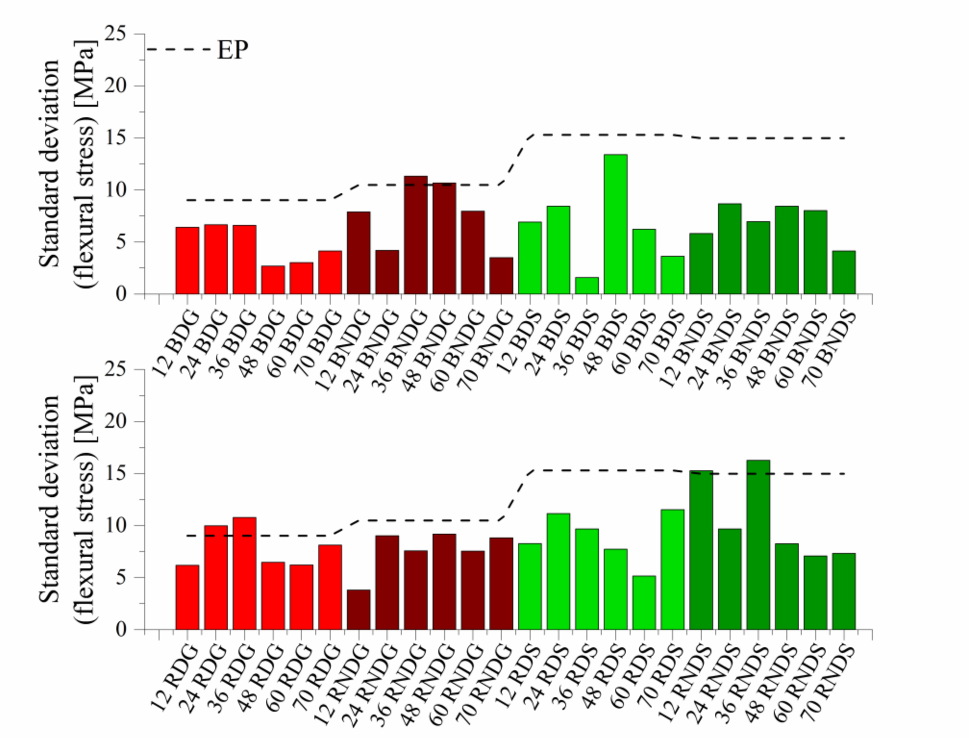

Figure S4. Standard deviation at the flexural stress for degassed and non-degassed composites containing base diatoms and rinsed diatoms.

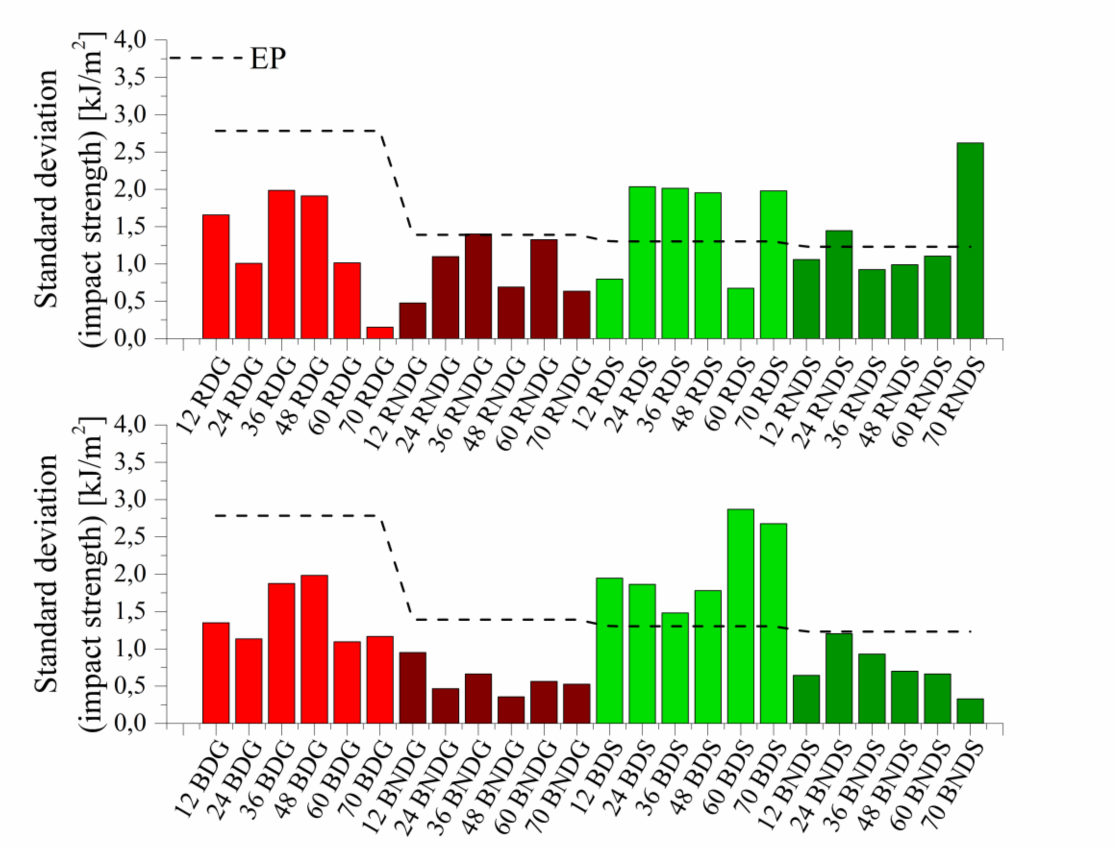

Figure S5. Standard deviation at the impact strength for degassed and non-degassed composites containing base diatoms and rinsed diatoms.
